# Solvent-Assisted Secondary Drying of Spray-Dried Polymers

**DOI:** 10.1007/s11095-020-02890-0

**Published:** 2020-07-31

**Authors:** Kimberly B. Shepard, April M. Dower, Alyssa M. Ekdahl, Michael M. Morgen, John M. Baumann, David T. Vodak

**Affiliations:** 1 Research & Development, Lonza Pharma, Biotech and Nutrition, Bend, Oregon USA; 2 Product Development, Lonza Pharma, Biotech and Nutrition, Bend, Oregon USA; 3 Department of Chemical Engineering, University of Texas at Austin, Austin, Texas USA

**Keywords:** amorphous solid dispersion, diffusion, eudragit L100, secondary drying, spray-drying

## Abstract

**Purpose:**

The purpose of this work is to introduce solvent-assisted secondary drying, a method used to accelerate the residual solvent removal from spray dried materials. Spray-drying is used to manufacture amorphous solid dispersions, which enhance the bioavailability of active pharmaceutical ingredients (APIs) with low aqueous solubility. In the spray-drying process, API and excipients are co-dissolved in a volatile organic solvent, atomized into droplets through a nozzle, and introduced to a drying chamber containing heated nitrogen gas. The product dries rapidly to form a powder, but small amounts of residual solvent (typically, 1 to 10 wt%) remain in the product and must be removed in a secondary-drying process. For some spray-dried materials, secondary drying by traditional techniques can take days and requires balancing stability risks with process time.

**Methods:**

Spray-dried polymers were secondary dried, comparing the results for three state-of-the-art methods that employed a jacketed, agitated-vessel dryer: (1) vacuum-only drying, (2) water-assisted drying, or (3) methanol-assisted drying. Samples of material were pulled at various time points and analyzed by gas chromatography (GC) and Karl Fischer (KF) titration to track the drying process.

**Results:**

Model systems were chosen for which secondary drying is slow. For all cases studied, methanol-assisted drying outperformed the vacuum-only and water-assisted drying methods.

**Conclusions:**

The observation that methanol-assisted drying is more effective than the other drying techniques is consistent with the free-volume theory of solvent diffusion in polymers.

**Electronic supplementary material:**

The online version of this article (10.1007/s11095-020-02890-0) contains supplementary material, which is available to authorized users.

## Introduction

Amorphous solid dispersions have been successfully used to improve the oral bioavailability of low aqueous solubility active pharmaceutical ingredients (APIs), which constitute the majority of compounds in today’s pharmaceutical pipelines (1–4).^.^ Spray-drying is one of many techniques to produce amorphous solid dispersions, along with hot melt extrusion, thermokinetic mixing, and others (5,6). Spray-drying has proven especially useful for APIs that are sensitive to high-temperature exposure and can be dissolved in a volatile solvent (7,8).

During a typical spray-drying process, API and excipients (e.g., polymers, surfactants, or stabilizing aids), are co-dissolved in a volatile solvent such as acetone, methanol, tetrahydrofuran (THF), or dichloromethane (DCM). The resulting spray solution is pumped through an atomizer, which converts the liquid to small droplets (on the order of microns to hundreds of microns). The atomized liquid is sprayed into a drying chamber, where it encounters hot drying gas. The solvent rapidly evaporates from the droplets, forming a solidified spray-dried dispersion (SDD) particle, which is typically amorphous (9).

This solvent-removal process from the SDD is limited both by kinetic and thermodynamic considerations. On the timescale of the spray-drying process, the solvent cannot fully diffuse out of the SDD particle. Additionally, solvent vapor is present in the dryer outlet stream, so even at equilibrium, some solvent would remain in the SDD. By adjusting spray-drying parameters—such as the spray-dryer outlet temperature (T_out_) or ratio of spray solution to drying gas—the amount of residual solvent can be reduced, but never fully eliminated (10).

Removal of residual solvent from SDDs to acceptable levels is critical for patient safety, as well as the physical and chemical stability of the SDD. For patient safety, International Council for Harmonisation (ICH) guidelines specify the maximum concentrations of residual solvents permissible in an oral pharmaceutical product, as well as a maximum total solvent intake per day for patients (11). When setting limits for residual solvents under ICH Option 1, for example, acetone content must be below 0.5% by weight, whereas methanol content must not exceed 0.3% by weight. The limits for other spray-drying solvents, such as THF and DCM, are even lower. In addition to these regulatory requirements, the physical stability must also be considered for these high-energy, metastable systems. A physically stable SDD must maintain a homogeneous, amorphous state during long-term storage (i.e., at least 2 years). The presence of solvent in an amorphous material has a plasticizing effect, introducing molecular mobility into the system (12). This increases the risk of the non-equilibrium amorphous material recrystallizing or phase separating. Residual solvent can also introduce chemical stability risks, including reactions with the API or with other excipients in the formulation, forming unwanted degradants.

To prevent these potential problems, secondary drying is used to remove residual solvent from SDDs after spray-drying. Two secondary drying methods are most common for pharmaceutical SDDs at manufacturing scale: (1) tray drying and (2) vacuum drying in an agitated mixer (13). In tray drying, so-called “wet” SDD (i.e., SDD that contains residual solvent before secondary drying) is spread into a thin (1- to 2-cm) layer on trays and placed in an environmental chamber. The temperature, humidity, and gas flow within the chamber are controlled. Tray drying is a batch process and requires extensive personal protective equipment to prevent operator exposure during loading and unloading of powder. As a result, tray drying is commonly used at small scales, but is not favored for large-scale manufacturing or the manufacture of SDDs using high-potency active compounds.

The second method, which uses agitated-vessel dryers, is more flexible, since equipment can accommodate early clinical-size batches (3 L) up to full commercial scale batches (5000 L) for pharmaceutical applications. Powder is loaded into an enclosed vessel and a sweep gas is flowed over the surface of the powder while an impeller rotates through the powder bed (14). Agitation is controlled to prevent fluidization of the powder. Typically, the vessel is run under vacuum with a low purge rate, using nitrogen as the purge gas to reduce the risk of explosion and avoid saturating the headspace of the drying vessel with residual solvent vapor. In this paper, we describe methods in which the purge gas is partially saturated with water (15) or methanol vapor to improve the drying kinetics of the process.

The kinetics of secondary drying are limited by two factors: (1) diffusion of solvent out of the solid particle (16) and (2) convection of solvent away from the solid particle. Convection and diffusion are coupled at the surface of the particle, where adequate convection drives the concentration gradient in the particle, enabling fast diffusion. In tray drying, convection limitations can be significant, so powder bed depths must be kept shallow (1 to 2 cm). In agitated vacuum-only drying, convection may or may not be limiting based on the material, scale, gas flow rate, and impeller settings. When spray-dried materials require secondary drying times on the order of days, diffusion is typically limiting; thus the experiments presented here are designed to focus on the diffusion-limited case.

The literature describes a great deal of work aimed at modelling the diffusion of solvent in an amorphous polymer. Vrentas and Duda pioneered the application of a free-volume theory of Fickian diffusion in amorphous polymers (17,18). Numerous subsequent studies by these authors and others have further developed these models and demonstrated methods to relate model parameters to experimental measurements (16,19,20). The model proposed in this study combines the approaches of Vrentas and Duda, as well as Sturm *et al* (16) and Schabel *et al* (21) to describe a ternary polymer-solvent-solvent system dried far below the glass-transition temperature (T_g_) of the spray dried polymer, where T_g_ changes with solvent concentration.

This study was focused on increasing the diffusion coefficient for secondary drying in agitated vacuum mixers in three configurations to determine which removed residual solvent fastest for three challenging spray dried powder compositions. Specifically, a jacketed, agitated vacuum dryer was used to secondary dry materials using methods termed (1) “vacuum-only drying,” (2) “water-assisted drying” (in which water vapor was added to the nitrogen purge gas to increase drying kinetics), and (3) “methanol-assisted drying^”^ (in which methanol vapor was added to the nitrogen purge gas to increase drying kinetics). In this study, the three methods were compared for three model systems known to have slow secondary drying. In all three systems, methanol-assisted drying method proved to be 2 to 5-fold faster than other secondary-drying techniques. A free-volume model is proposed to explain the faster kinetics of methanol-assisted drying relative to the other secondary-drying techniques.

## Materials and Methods

### Materials

Poly(methyl methacrylate-co-methacrylic acid), or PMMAMA (trade name Eudragit® L100), was purchased from Evonik (Essen, Germany). Cellulose acetate phthalate (CAP) was purchased from Eastman Chemical Company (Kingsport, Tennessee, USA). Solvents used for spray-drying and secondary drying (i.e., acetone, THF, methanol) were purchased from Pharmco (Toronto, Ontario, Canada) and Honeywell International Inc. (Charlotte, North Carolina, USA).

### Methods

This section describes the manufacture of spray-dried materials, the experimental setup and methods used for secondary-drying trials, and the following analytical methods, which were used to characterize the samples: differential scanning calorimetry (DSC), process mass spectroscopy, gas chromatography (GC), Karl Fischer (KF) titration.

### Manufacture of Spray-Dried Material

Spray-dried polymers were manufactured on a custom-built clinical-scale spray dryer with a nominal gas flow rate of 150–200 kg/h. The atomizing nozzle used was a Spraying Systems pressure-swirl nozzle (Model SK-80-16) (Spraying Systems Co., Wheaton, Illinois, USA). The spray-solution composition and spray-drying parameters are summarized in Table I. (Unless otherwise specified, all compositions referenced in this paper are in weight percent.)

**Table I:** Spray Solution Composition and Spray-Dryer Parameters for Manufacture of Study Materials

Sample	Value		
	100% PMMAMA From Acetone	100% PMMAMA From THF	100% CAP From THF
Spray-solution composition	6% solids in 97/3 acetone/water	6% solids in 97/3 THF/water	5% solids in 97/3 THF/water
Drying-gas flow rate, g/min	1850		
Inlet temperature (T_in_), °C	99	96	120
Outlet temperature (T_out_), °C	35	35	45
Spray-solution flow rate, g/min	156	177	184
Atomization pressure, psi	400		323
Batch size, g solids	2000	2000	750

“Wet” spray-dried materials were collected via cyclone and refrigerated until used.

### Secondary-Drying

#### Experimental Setup for Secondary Drying

An Ekato VPT3 agitated dryer (Ekato Corporation, Oakland, New Jersey, USA) was used for all secondary drying experiments. The working fill volume of the vessel was 1–3 L, so experiments could be performed over a variety of conditions using little material.

The standard Ekato VPT setup (see Supporting Information) was used for vacuum-drying experiments. Dry nitrogen was fed into the vessel and the vessel jacket heated the material to a target temperature. Using a vacuum pump, a pressure of about 30–50 mbar was maintained in the vessel. During sampling events, the vacuum in the vessel was briefly broken so that the lid could be temporarily removed and a sample taken before resuming vacuum and normal operation.

**Fig. 1:**
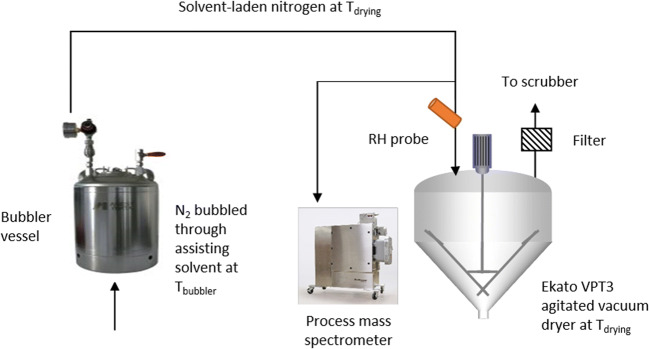
Modified Ekato VPT agitated vacuum dryer set up for water- or methanol-assisted secondary drying.

For the water- and methanol-assisted drying trials, the Ekato VPT3 was modified to incorporate water or methanol vapor (Fig. 1) into the purge gas. A dry nitrogen stream was fed through the bottom of a bubbler vessel that saturated the nitrogen with the assisting solvent. The temperature of the bubbler (T_bubbler_) was adjusted to achieve the target mass fraction of solvent in the nitrogen stream. The target mass fraction of solvent was dependent on the desired relative humidity (%RH for water-assisted drying) or relative saturation (%RS for methanol-assisted drying) in the chamber. The incoming gas stream was heated to the drying-vessel temperature (T_drying_) prior to introduction.

Immediately before entering the drying vessel, the nitrogen stream was sampled with a temperature probe and either an RH probe (for water-assisted methods) or a process mass spectrometer (for methanol-assisted methods). The mass spectrometer allowed real-time assessment of the methanol mass fraction in the nitrogen stream, which enabled calculation of the RS in the chamber. The T_bubbler_ value was adjusted to alter the mass fraction of solvent in the nitrogen stream based on the RH probe or the mass spectrometer data. During the trials, the bubbler ran at approximately 85% to 95% efficiency.

Rather than using a vacuum pump to achieve a vacuum in the vessel during solvent-assisted operation, a scrubber system was used to vent the vessel and maintain sub-ambient pressure (0.8 to 0.9 bar). Before opening the vessel for sampling or material discharge, a dry nitrogen sweep was introduced for 1 min to ensure no condensation occurred when room-temperature air was introduced to the system.

#### Secondary-Drying Experiments

For agitated-dryer experiments, the customized system described above was used in three configurations: vacuum-only drying, water-assisted drying, and methanol-assisted drying. Process conditions for the agitated-dryer experiments are provided in Table II. The processing temperature was 50°C for all experiments. Depending on the powder density (~0.2–0.3 g/cm^3^), 300 to 400 g of material was loaded into the vessel to achieve fill volume of >30%. Agitating paddle speeds were set to 100 rpm.

**Table II:** Agitated-Dryer Process Parameters for Secondary-Drying Experiment DSC Analysis

Experiment No.	Test type	Value				
		Purge gas	Purge-gas flow rate (SCFH*)	Chamber pressure (mbar)	Residual solvent	Material
1	Vacuum-only	Dry N_2_	1.2	50	Acetone	PMMAMA
2	Water-assisted	N_2_ and water, 50%RH	15 to 20	850 to 900	Acetone	PMMAMA
3	Methanol-assisted	N_2_ and methanol, 20%RS	15 to 20	850 to 900	Acetone	PMMAMA
4	Methanol-assisted	N_2_ and methanol, 20%RS	20	850 to 900	Acetone	PMMAMA
5	Methanol-assisted	N_2_ and methanol, 20%RS	4	850 to 900	Acetone	PMMAMA
6	Methanol-assisted	N_2_ and methanol, 10%RS	20	850 to 900	Acetone	PMMAMA
7	Vacuum-only	Dry N_2_	1.2	50	THF	PMMAMA
8	Water-assisted	N_2_ and water, 50%RH	15	850 to 900	THF	PMMAMA
9	Methanol-assisted	N_2_ and methanol, 20%RS	15	850 to 900	THF	PMMAMA
10	Vacuum-only	Dry N_2_	1.2	40	THF	CAP
11	Water-assisted	N_2_ and water, 45%RH	15	850 to 900	THF	CAP
12	Methanol-assisted	N_2_ and methanol, 20%RS	15	850 to 900	THF	CAP

*SCFH: Standard cubic foot per hour

Plasticization occurs when an amorphous material absorbs solvent, introducing molecular mobility to the polymer matrix and decreasing the material’s T_g_, which is measured on heating. DSC was used to analyze the T_g_ of the PMMAMA in three different solvents—acetone, methanol, and water—to measure how each solvent affected the T_g_ (and, thus, molecular mobility) of the polymer.

PMMAMA was used as received from the manufacturer and then dried in a low-humidity chamber (RH < 5%, ambient temperature) for 3 days. Dried PMMAMA (2- to 5-mg) samples were weighed into Tzero pans (TA Instruments, New Castle, Delaware, USA) and equilibrated in one of the following ways. Samples designated 0%RH or 0%RS were hermetically sealed in Tzero pans inside the low-humidity chamber to prevent the ingress of moisture. Samples for T_g_-*versus*-%RH analysis were equilibrated in adjustable controlled-humidity boxes (at 50% and 75%RH) at ambient temperature overnight, and hermetically sealed in Tzero pans before removal. Samples for T_g_-*versus*-%RS analysis were tared and then placed in a sealed chamber with an open vial of solvent. Samples were removed one at a time after different exposure intervals (5 min – 4 h) and immediately sealed. The mass uptake of solvent was measured by weighing the samples after sealing. The midpoint T_g_ of all samples was measured using a TA instruments Q2000 DSC instrument, scanning in modulated DSC mode from 0°C to 150°C at a rate of 2.5°C/min, with 1.5°C amplitude modulation and a period of 60 s.

**Fig. 2:**
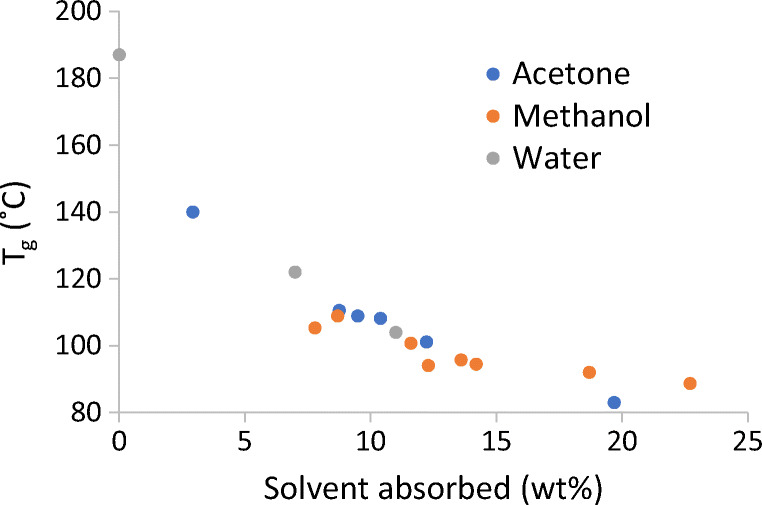
Glass transition temperature of PMMAMA polymer with absorbed solvents.

The results of these measurements are shown in Fig. 2. For wet PMMAMA samples containing <3% solvent by weight, the T_g_ is too high to be measured using a hermetic pan, because the hermetic seal typically fails at ~140°C (or 2-bar internal pressure). Data show that while PMMAMA was strongly plasticized by all three solvents (shown by an ~50°C decrease in T_g_ at 7% solvent concentration), the T_g_ was still >100°C for all solvent concentrations under 10%. The degree of plasticization was similar among the three solvents when compared on a mass basis.

### Mass Spectrometry

For methanol-assisted drying, the methanol mass fraction in the ingoing nitrogen stream was quantified using a ProMaxion process mass spectrometer (Ametek Process Instruments, Pittsburgh, Pennsylvania, USA). The equipment was calibrated for nitrogen, oxygen, argon, carbon dioxide, and methanol, using dry air saturated with methanol at a known temperature.

### GC Headspace

The concentrations of residual solvent (from spray-drying) and assisting solvents (from secondary drying) were quantified using GC headspace analysis. A known quantity of material was dissolved in 4 mL of dimethylacetamide (DMAC) to liberate solvent contained in the solid. An Agilent G7890 GC (Agilent Technologies, Santa Clara, California, USA) equipped with a flame ionization detector and split injection capability for capillary column operation was used with an Agilent 7697 automated headspace sampler. The column used was an Agilent DB-624: 30 m × 0.32 mm interior diameter (ID) × 1.8 μm. Detailed experimental parameters are given in the Supporting Information. Peaks were quantitated and compared with a standard curve of known concentrations. In the method, the following approximate retention times were observed: methanol, 2.6 min; acetone, 4.2 min; DCM, 5.0 min; THF, 8.3 min; DMAC, 12.4 min.

### KF Titration

Water content was quantified using a coulometric Metrohm 851® Titrando KF oven titrator (Metrohm USA Inc., Tampa, Florida, USA), with the generator electrode operated in diaphragm mode. Hydranal™-AK anolyte solution, CG-K catholyte solution, and Hydranal water standard (Fluka™) were used (Honeywell International Inc. Charlotte, North Carolina, USA). A 20- to 100-mg sample of each replicate was sealed into a KF vial. The temperature for standards was 150°C and the sample temperatures ranged from 150°C to 180°C.

## Results

In this section, we present results of secondary-drying trials with spray dried polymers, focused on the removal of (1) residual acetone from PMMAMA, (2) residual THF from PMMAMA, and (3) residual THF from CAP. Three secondary-drying techniques were examined: (1) vacuum-only drying, (2) water-assisted drying, and (3) methanol-assisted drying.

### Secondary Drying of Spray-Dried PMMAMA with Residual Acetone

In this section (Experiments 1 through 3, see Table II), the three secondary-drying techniques were used to remove residual solvent from PMMAMA sprayed from acetone on a clinical-scale dryer. The PMMAMA contained approximately 10% residual solvent. It is often difficult to remove residual acetone from spray-dried PMMAMA to below the ICH limit of 0.5%, typically requiring multiple days of drying at aggressive conditions (data not shown). Thus, clear need exists for development of an improved, efficient secondary-drying protocol that can reduce levels of residual acetone on a time scale of hours, rather than days.

**Fig. 3:**
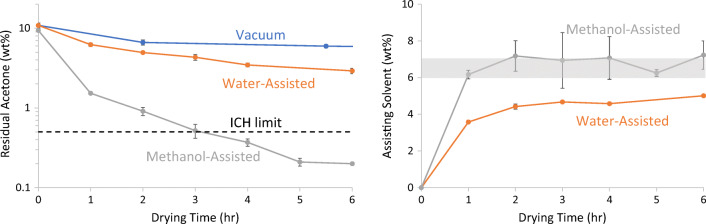
Secondary drying for PMMAMA with residual acetone using three techniques (left), and uptake of assisting solvent (right). The grey shaded area indicates the target concentration ± 0.5% for assisting solvent absorbed during the experiment.

Figure 3 shows drying curves for removal of acetone from spray-dried PMMAMA for the three techniques. All drying experiments were conducted at 50°C. The T_g_ of the wet polymer (i.e., before secondary drying) was at least 50°C higher than the drying temperature, which means the material was in the glassy state during drying. As the figure shows, methanol-assisted drying removed acetone significantly faster than other techniques, reducing the acetone concentration to the ICH limit in approximately 3 h. No assisting solvent was used in vacuum-only drying, so this technique was the slowest, failing to reach the ICH limit even after 16 h (4.7% residual acetone remained at 16 h).

Water-assisted drying was conducted at 50%RH and methanol-assisted drying was conducted at 20%RS. These values were chosen to target a solvent uptake of 6% to 7% for water and methanol [see dynamic vapor sorption (DVS) data in Supporting Information]. As the results in Fig. 2 show, the T_g_ of PMMAMA was expected to be similar, using methanol or water as the assisting solvent. Figure 3 shows the uptake of assisting solvent (water or methanol) over time during the drying experiments. With methanol-assisted drying in Experiment 3, the target amount of methanol (shaded area) was rapidly absorbed and the methanol content remained in that range for the duration of the experiment. With water-assisted drying in Experiment 2, the target sorption of water was not reached. The limited absorption of water in the polymer was attributed to the low humidified purge-gas flow rate. When the same material was secondary-dried in a humidified tray dryer with a high purge-gas turnover, the target sorption of water was reached (data not shown). A set of experiments to probe the effect of sweep-gas flow rate and relative saturation of the assisting solvent was performed on the PMMAMA/acetone system (Experiments 4–6, see Table II). The full results are given in the Supporting Information.

Once the residual-solvent content from secondary-drying is below ICH limits, the assisting methanol must be removed from the material. Methanol, which has an ICH limit of 0.3%, is efficiently removed from most spray-dried systems. To demonstrate this, at the conclusion of the 6-h methanol-assisted drying experiment, the sweep gas was switched to 15%RH water at 50°C. After 3 h of drying, the methanol content was reduced from 5% to 0.7%. Slightly more aggressive drying at higher humidity, or 2 more hours of drying time, would likely reduce the methanol content below the ICH limit. Methanol removal using vacuum-only drying is also feasible. Although water does not need to be removed for regulatory reasons, SDDs are often packaged with desiccant to maintain specified water activity, because dry-powder storage of SDDs is preferred to improve stability.

### Secondary Drying of Spray-Dried PMMAMA with Residual THF

**Fig. 4:**
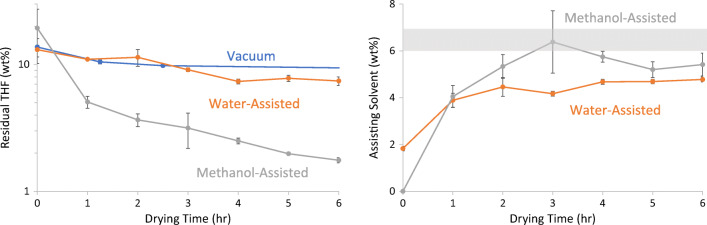
Secondary drying for PMMAMA with residual THF using three techniques (left, and uptake of assisting solvent (right). The grey shaded area indicates the target concentration ± 0.5% for assisting solvent absorbed during the experiment. ICH limit is 0.076%, not shown.

In this work (Experiments 7 through 9, see Table II), the three secondary-drying techniques were used to remove residual solvent from PMMAMA sprayed from THF. As with acetone, past experience has shown that removal of residual THF after spray-drying to the ICH specification of 0.076% is difficult. The PMMAMA spray-dried on a clinical-scale dryer from THF contained high levels of residual solvent, ranging from 12% to 20%. To meet the ICH THF specification of 0.076%, it would be necessary to reduce the solvent concentration by more than 2 orders of magnitude. As with acetone removal, the methanol-assisted drying reduced the THF content significantly faster than the other techniques, as shown in Fig. 4. Again, vacuum-only drying was the slowest, with water-assisted drying falling in the middle. However, the residual THF in the methanol-assisted case remained far above ICH limits at 6 h. If the log-linear drying trajectory for methanol-assisted drying is extrapolated using the 0–6 h data, it will reach the ICH limit in approximately 20 h, compared to 50+ hours for water-assisted drying. Increasing the temperature or methanol %RS further in future experiments could likely improve performance further.

In these experiments, the target concentration for the assisting solvent was again 6% to 7%, but both methanol and water contents fell short of this value, at 4.7% and 5.5%, respectively at the end of the experiment. The discrepancy in solvent absorption is likely explained by the presence of large amounts of residual THF, altering the equilibrium. This was not experimentally verified by DVS, however, due to the instrument’s incompatibility with THF.

### Secondary Drying of Spray-Dried CAP with Residual THF

In this work (Experiments 10 through 12, see Table II), the three secondary-drying techniques were used to remove residual solvent from CAP sprayed from THF. The CAP contained approximately 10% residual THF. The wet T_g_ of the CAP remained at or above 100°C for these experiments (measured by DSC, data not shown), so the CAP was in the glassy state during secondary drying.

**Fig. 5:**
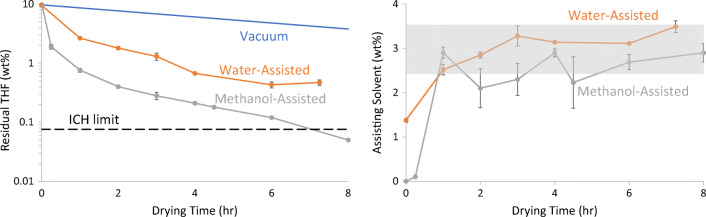
Secondary drying of CAP with residual THF using three techniques (left), and uptake of assisting solvent (right). The grey shaded area indicates the target concentration ± 0.5% for assisting solvent absorbed during the experiment.

Results for the three secondary drying trials are shown in Fig. 5. Again, methanol-assisted drying had the fastest drying kinetics, reducing residual solvent levels below the ICH limit of 0.076% within 8 h. Water-assisted drying was second-fastest, and vacuum-only drying was slowest. Figure 5 also shows the uptake of assisting solvent during drying. The target concentration of assisting solvent was 3%. This concentration was limited by a practical reason; only 3% water was absorbed into the CAP at 50%RH at 50°C, and exceeding this RH value led to condensation when the material was sampled at ambient temperature. This highlights a practical advantage of methanol-assisted drying that is covered in detail in the Discussion section. If needed, more aggressive drying conditions can be pursued using methanol as an assisting solvent instead of water due to methanol’s higher volatility. Both water- and methanol-assisted cases reached the target assisting solvent concentration, though the methanol levels were slightly lower than the water levels (Fig. 5, right). Even though less assisting solvent was used, methanol-assisted drying outperformed water-assisted drying.

## Discussion

### Practical Advantages of Methanol-Assisted Drying

**Fig. 6:**
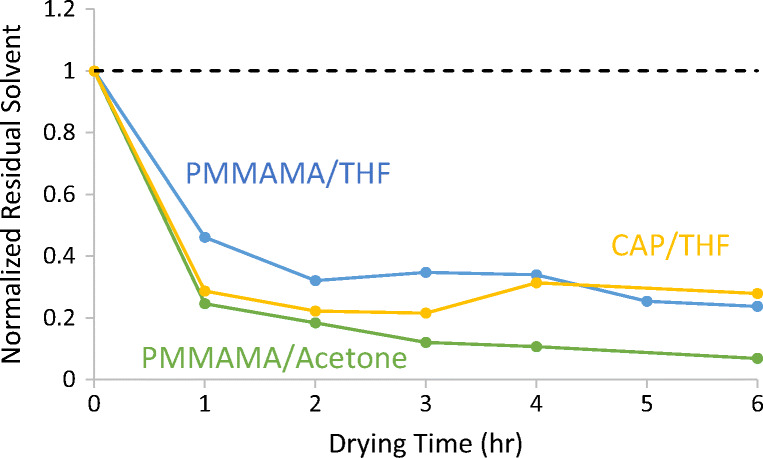
Normalized residual solvent (concentration of residual solvent during methanol-assisted drying divided by concentration of residual solvent during water-assisted drying) for the three polymer/solvent systems studied. Dotted line represents equal performance between methanol-assisted and water-assisted drying.

This study has shown that methanol-assisted secondary drying significantly improves drying kinetics compared to vacuum-only or water-assisted drying for the range of polymers and spray solvents studied here. To summarize the generality of secondary-drying improvement via methanol-assisted drying, Fig. 6 shows an overlay of the drying results for the three polymer/solvent systems. In this figure, the residual-solvent concentration achieved during methanol-assisted drying is divided by the residual-solvent concentration during water-assisted drying at each time point. For values of less than one, methanol-assisted drying is faster than the water-assisted drying. The figure demonstrates that from the first time point on, methanol-assisted drying is significantly faster than water-assisted drying for all three polymer/solvent systems studied.

From an equipment and process design perspective, use of methanol as an assisting solvent has additional advantages. Due to its increased volatility and polymer affinity, a lower relative saturation level (%RS) is needed to achieve equivalent mass of absorbed solvent compared to water-assisted drying. This reduces the risk of condensation during sampling and unloading the drying vessel, or anywhere in the system at risk of heat loss. Additionally, higher levels of absorbed solvent are feasible for the more-volatile assisting methanol, leading to further potential increases in drying kinetics. For secondary drying of water-sensitive compounds methanol-assisted drying is especially advantageous because exposure to water can be minimized.

The main safety considerations for a methanol-assisted process are easily addressed. Risks due to the use of a flammable solvent or fluidized powder are reduced by system design, use of nitrogen as a purge gas, and monitoring the chamber oxygen concentration during processing. The residual methanol can be removed through standard secondary drying, to meet ICH limits and prevent toxicity concerns. These additional considerations are acceptable for challenging drying systems where acceleration of the secondary-drying process is necessary for scale-up or commercialization feasibility.

### Mechanism for Improved Drying with Methanol as Assisting Solvent

The diffusion of solvents through amorphous polymers often follows Fickian diffusion, which is governed by the free volume in the system (16). In the three systems studied here, secondary drying is conducted at conditions deep in the glassy state—that is, more than 40°C below the wet T_g_ of the polymer. When methanol rather than water is used as the assisting solvent for secondary drying, residual-solvent removal is faster, even when equal masses of assisting solvent are absorbed. This result implies that the diffusion coefficient of the residual solvent is higher when the polymer contains assisting methanol than assisting water.and the diffusion coefficient is even higher compared to a system where no assisting solvent is present (e.g., vacuum-only drying).

In vacuum-only drying (i.e. a single-solvent system), the size of free-volume pockets is as described in Sturm *et al*. (16) The model accounts for a decreasing diffusion coefficient as the sample becomes drier and less plasticized. In solvent-assisted drying, using water or methanol, the assisting solvent content stays constant throughout the secondary-drying process. Thus, the diffusion coefficient decreases less over time and residual solvent removal does not slow down as drastically.

There is experimental and theoretical precedence that inclusion of an assisting solvent increases the available free volume in the system, increasing the diffusion coefficient of the residual solvent. Schabel *et al*. (21) measured the diffusion coefficient of a ternary polymer-solvent-solvent system using the free-volume theory of Vrentas *et al*. (22) In the ternary system, the two solvents had a large impact on each other’s diffusion coefficient. The presence of one solvent increased the diffusion coefficient of the second solvent, and vice versa. In a second publication, Schabel *et al*. showed drying curves for a ternary methanol-toluene-poly(vinyl acetate) system and proposed a framework to account for an additional solvent into the calculation of the diffusion coefficient (23). In this case, the methanol dried much more rapidly than the toluene. Once the methanol was evaporated, the drying kinetics of the toluene slowed down drastically, suggesting that the methanol acted as a de facto assisting solvent until its depletion.

### Diffusion Coefficient Calculations in Ternary Systems Relevant to Solvent-Assisted Secondary Drying

As discussed above, methanol is a better assisting solvent for the polymer and solvent systems tested, compared with water at equal mass fractions. To consider the ternary drying system presented here, where the polymer is deep in the glassy state, the theoretical approaches used by Sturm *et al*. and Schabel *et al*. must be combined. Sturm *et al*. modified the binary free-volume model to account for the changing T_g_ of the system as it is dried, when the drying temperature is below the T_g_, as is the case for our system. Schabel *et al*. used the Vrentas free-volume model with two solvents and one polymer, but with a constant polymer T_g_.

In modeling our system, the self-diffusion coefficient expressions, shown in Eqs. 1 and 2, are unchanged from Schabel *et al*.:

1 $$ {D}_1={D}_{01}\cdotp \mathit{\exp}\left(-\frac{\omega_1{V}_1^{\ast }+{\xi}_{1P}/{\xi}_{2P}\cdotp {\omega}_2{V}_2^{\ast }+{\xi}_{1P}{\omega}_P{V}_P^{\ast }}{\frac{V_{FH}}{\gamma }}\right)\  and $$

2 $$ {D}_2={D}_{02}\cdotp \mathit{\exp}\left(-\frac{\omega_1{V}_1^{\ast }{\xi}_{2P}/{\xi}_{1P}+{\omega}_2{V}_2^{\ast }+{\xi}_{2P}{\omega}_P{V}_P^{\ast }}{\frac{V_{FH}}{\gamma }}\right), $$

with the expression for the amorphous material free-volume term in the denominator (*V*_*FH*_/*γ*) defined using Sturm’s method for expressing the polymer free volume:

3 $$ {V}_{FH}/\gamma ={\omega}_1\left({K}_{I,1}/{\gamma}_1\right)\left[\left({K}_{II,1}-{T}_{g,1}\right)+T\right]+{\omega}_2\left({K}_{I,2}/{\gamma}_2\right)\left[\left({K}_{II,2}-{T}_{g,2}\right)+T\right]+{\omega}_P\left({K}_{I,P}/{\gamma}_P\right)\left[{K}_{II,P}-\left(1-\lambda \right)f\left({\omega}_1,{\omega}_2\right)+\lambda \left(T-{T}_{g,P}\right)\right] $$

In these equations, Component 1 is the residual solvent, Component 2 is the assisting solvent, and Component P is the polymer. The variables are defined as follows: ω_i_ is the mass fraction of Component i; V_i_* is the specific volume of the jumping unit for Component i; ξ_iP_ is the ratio of the molar volume of the jumping unit of i to the molar volume of the polymer jumping unit, P (ξ_ij_ = ${V}_{\mathrm{i}}^{\ast }{M}_i/{V}_P^{\ast }{M}_P$) (21,24); V_FH_/γ is the free volume of the amorphous phase with λ describing the nature of the change in volume contraction at T_g_ (25); K_I,i_/γ_i_ and K_II,i_ are the free-volume (WLF) parameters for Component i; T_g,i_ is the T_g_ of component i; f(ω) is the T_g_ depression from pure polymer caused by the presence of solvent; and D_0i_ is the pre-exponential factor.

For this work, as in Sturm *et al*., the Gordon-Taylor equation is used to determine the T_g_ of the wet polymer (24). For a ternary system, the Gordon-Taylor equation can be extended as follows:

4 $$ f\left(\omega \right)={T}_{g,P}-\frac{\omega_1{T}_{g1}+{\omega}_2{T}_{g2}{k}_{GT,1P}/{k}_{GT,2P}+\left(1-{\omega}_1-{\omega}_2\right){T}_{gP}{k}_{GT,1P}}{\omega_1+{\omega}_2{k}_{GT,1P}/{k}_{GT,2P}+\left(1-{\omega}_1-{\omega}_2\right){k}_{GT,1P}}, $$

where k_GT,iP_ is the Gordon-Taylor parameter for the binary system of solvent i and polymer P. The combination of calculations and experiments necessary to define all the constants in the above equations are summarized in a review by Danner (26).

From this model, the improved experimental performance of solvent-assisted drying compared with vacuum drying can be justified. The presence of an assisting solvent increases the free-volume present in the system (Eq. 3), thereby increasing the diffusion coefficient. An assisting solvent contributes predominantly to the free volume via the term (*K*_*I*, 2_/*γ*_2_)[(*K*_*II*, 2_ − *T*_*g*, 2_) + *T*]. The free volume contribution of the assisting solvent from the (*K*_*I*, 2_/*γ*_2_)[(*K*_*II*, 2_ − *T*_*g*, 2_) + *T*] term is positive for T > (K_II,2_ – T_g,2_). This term is positive at temperatures relevant to secondary drying (> 200 K) for all 46 solvents reported in Hong’s work (24). It would therefore be expected that the presence of nearly any assisting solvent – water, methanol or otherwise – would likely increase the diffusion coefficient of the residual solvent compared to no assisting solvent.

This model can also be applied to the water-assisted and methanol-assisted drying experiments described above in a qualitative manner without direct knowledge of many of the coefficients. For calculation of D_1_ with constant residual solvent and polymer, only the following parameters change between methanol-assisted and water-assisted cases: K_I,2_/γ_2_, K_II,2_-T_g2_, V^*^_2_ (ξ_2P_/ξ_1P_), and f(ω). The first two of these parameters—K_I,2_/γ_2_, K_II,2_-T_g2_—are solvent properties, reported in the literature. Physically, these WLF parameters are related to the deviation from Arrhenius behavior as the material is cooled through its glass transition (27). When the ratio ξ_2P_/ ξ_1P_ is taken, the contribution of the polymer jumping volume cancels out, so the ratio is dependent only on solvent molar volume properties. The f(ω) parameter depends on how strongly a solvent plasticizes the polymer. Increases in K_I,2_/γ_2_ and K_II,2_-T_g2_ lead to increases in D_1_. On the other hand, an increase in ξ_2P_/ξ_1P_ decreases D_1_.

A summary of the relevant parameters for comparing methanol-assisted and water-assisted drying cases is in Table III. The expression for the assisting solvent’s contribution to the hole free volume is ω_2_(K_I,2_/γ_2_)*(K_II,2_-T_g2_ + T). This is term 2 of eq. 3. For methanol, the value of (K_I,2_/γ_2_)*(K_II,2_-T_g2_ + T) is greater than that of water for 0 < T < 480 K. In the range of temperatures relevant to secondary drying (~300–340 K), it is 14–23% higher. Thus, the contribution of this term will always drive the diffusion coefficient higher for methanol than water. The expression for the assisting solvent’s contribution to the jumping unit volume is $$ {\xi}_{1P}/{\xi}_{2P}\cdotp {\omega}_2{V}_2^{\ast }=\raisebox{1ex}{${\omega}_2{V}_1^{\ast }{M}_1$}\!\left/ \!\raisebox{-1ex}{${M}_2$}\right. $$(24). This is term 2 in the numerator of eq. 2. This term is always lower for methanol than for water, also driving the diffusion coefficient higher for methanol than water. The impact of f(ω) on the free volume is more complex, and depends on the specific values of the polymer WLF parameters and the experimental temperature. However, for the polymers studied here, the plasticization effect of water and methanol on the polymer is similar, thus f(ω) has no impact when comparing methanol- and water-assisted secondary drying.

**Table III:** Expressions and Parameters for Comparison of Methanol-Assisted and Water-Assisted Drying of a Residual Solvent from a Polymer

	Methanol	Water
(K_I,2_/γ_2_)*(K_II,2_-T_g2_ + T)^a^	0.00177 (T-76.6)	0.00218 (T-152.3)
$$ \raisebox{1ex}{${V}_1^{\ast }{M}_1$}\!\left/ \!\raisebox{-1ex}{${M}_2$}\right. $$	0.0312 V^*^_1_ M_1_	0.0555 V^*^_1_ M_1_

^a^Solvent parameters from Sturm *et al*. (21) (All units in cubic centimeter per gram)

Comparing this theory to our experimental results, Fig. 2 demonstrated that the water and methanol reduce the T_g_ of PMMAMA similarly. Figures 3 and 4 then confirmed that methanol-assisted secondary drying increased the residual solvent diffusion coefficient more than water-assisted, resulting in faster residual solvent removal for both acetone and THF. Water and methanol also similarly reduce the T_g_ of CAP, resulting in the improved drying kinetics seen in Fig. 5. Thus the free-volume theory is in good agreement with the experimental results presented here.

## Conclusions

Use of methanol as an assisting solvent during secondary drying increases residual solvent-removal kinetics. This method is advantageous for high-T_g_ polymers and formulations for which secondary drying is slow by conventional means. In all cases examined in this study, methanol-assisted drying in an agitated dryer was significantly faster than comparable water-assisted drying or vacuum-only drying. This was explained through application of the free-volume theory as applied to a ternary polymer-solvent-solvent system. Solvent-assisted secondary drying is therefore an enabling approach for SDD manufacturing of materials that pose secondary drying challenges in the pharmaceutical arena.

## Electronic supplementary material

ESM 1 (PDF 406 kb)

## Abbreviations

API: Active pharmaceutical ingredient
CAP: Cellulose acetate phthalate
DMAC: Dimethylacetamide
DSC: Differential scanning calorimetry
DVS: Dynamic vapor sorption
GC: Gas chromatography
HPMCAS: Hydroxypropyl methylcellulose acetate succinate
ICH: International Conference on Harmonization
ID: Inner diameter
KF: Karl Fischer
PMMAMA: Poly(methyl methacrylate-co-methacrylic acid), trade name Eudragit® L100
RH: Relative humidity
RS: Relative saturation
SCFH: Standard cubic feet per hour
SDD: Spray-dried dispersion
SEM: Scanning electron microscopy
THF: Tetrahydrofuran
T_bubbler_: Temperature of the bubbler vessel
T_drying_: Temperature of the drying vessel
T_g_: Glass-transition temperature
T_in_: Inlet temperature
T_out_: Outlet temperature
