# Supplementary material for: Solvent-Assisted Secondary Drying of Spray-Dried Polymers
Source: Pharm Res. 2020 Jul 31;37(8):156. doi: 10.1007/s11095-020-02890-0 (PMC7395053; doi:10.1007/s11095-020-02890-0)
Supplement: Supplementary file 1 — (PDF 406 kb) [file 11095_2020_2890_MOESM1_ESM.pdf]

Supporting Information

Agitated Vessel Setup in Vacuum Mode

A schematic for typical setup of the agitated vessel in vacuum mode is shown below in Figure S1.

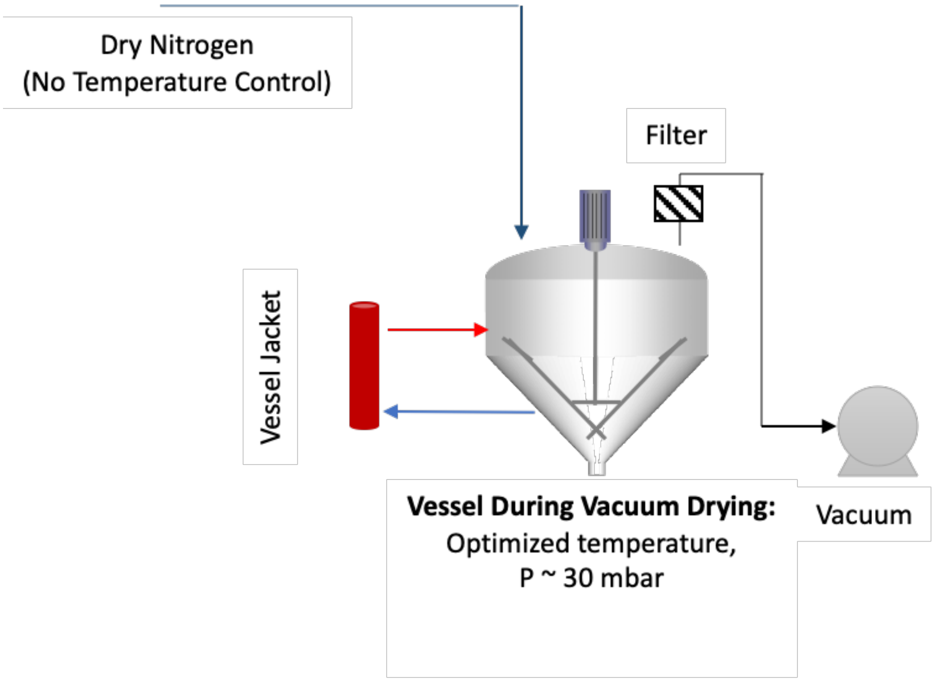

Figure S1. Standard Ekato VPT setup for vacuum secondary-drying experiments

Gas Chromatography (GC) and Headspace Autosampler Parameters

Detailed GC and headspace autosampler parameters are given in Table S1.

Table S1. GC and Headspace Autosampler Parameters

Parameter	Value
**GC Parameters**	
Lit offset	0.5 pA
Injector temperature	180°C
Detector temperature	260°C
Oven program	40°C for 5 min, 2°C/min to 45°C, hold at 45°C for 0 min, 30°C/min to 225°C, hold at 225°C for 2 min
Carrier gas	Hydrogen
Hydrogen flow rate	38mL/min
Compressed-air flow rate	400mL/min
Makeup-gas flow rate (N_2)	30mL/min
Carrier flow rate	1.6mL/min
Split ratio	9.4:1
**Headspace Autosampler Parameters**	
Oven temperature	105°C
Loop temperature	110°C
Transfer-line temperature	115°C
GC cycle time	25 min
GC injection time	1 min
Sample loop	1mL
Loop equilibration time	0.05 min
Loop fill time	0.2 min
Pressurization time	0.33 min
Pressurization gas	Nitrogen
Vial equilibration time	30 min
Carrier pressure	~9 psi
Vial pressure	~20 psi

DVS Data For Assisted Solvent Sorption

Sorption data was collected using DVS (DVS Advantage, Surface Measurement Systems, Allentown, Pennsylvania, USA) for a 5- to 10-mg sample of as-received PMMAMA polymer (Eudragit L100, Evonik). The sample was pre-dried in the DVS Advantage instrument at 60˚C for 1 hour. At each step in the P/P_0 ramp, the sample mass was allowed to stabilize for 6 to 12 hours. Sorption data for water and methanol are shown in Table S2.

Table S2. DVS sorption data for water and methanol in PMMAMA

Water		Methanol	
**% P/Po**	**Sorption**	**% P/Po**	**Sorption**
0.0	0.00	0.0	0.00
10.0	1.64	10.0	3.08
20.0	2.68	20.0	5.53
30.0	3.71	30.0	7.73
40.0	4.82	40.0	10.03
50.0	6.29	50.0	12.46
60.0	8.00	60.0	14.95
70.0	10.05	70.0	17.49
80.0	12.41	80.0	20.52
90.0	15.67	90.0	23.58

Supplementary Experiments With Solvent-Assisted Secondary Drying

To determine the robustness of the methanol-assisted drying method, this study examined the effect of sweep-gas flow rate and sweep gas %RS on the drying kinetics for the PMMAMA/acetone system. At larger scale, the ratio of sweep gas to powder is often lower to prevent powder fluidization. Based on DVS data (above), 3% methanol was expected to be absorbed into the powder at 10%RS, compared with 6% at 20%RS. (Experiments 4-6, Table 2) A comparison of the drying kinetics for control, low flow rate and low %RS conditions is shown in **Figure S2**. Reducing the sweep-gas flow rate, and therefore the supply of methanol vapor to the system, slowed down drying slightly compared to the control. In addition, it

reduced convective mass transfer. However, the endpoint residual acetone content was not significantly different. Reducing the relative saturation (10% RS versus 20% RS) greatly slowed drying compared to the control. The uptake of methanol during these drying experiments is shown in Figure S2. Methanol uptake in the control nearly matched the expected 6% and, in the low %RS case, it was close to the expected 3%.

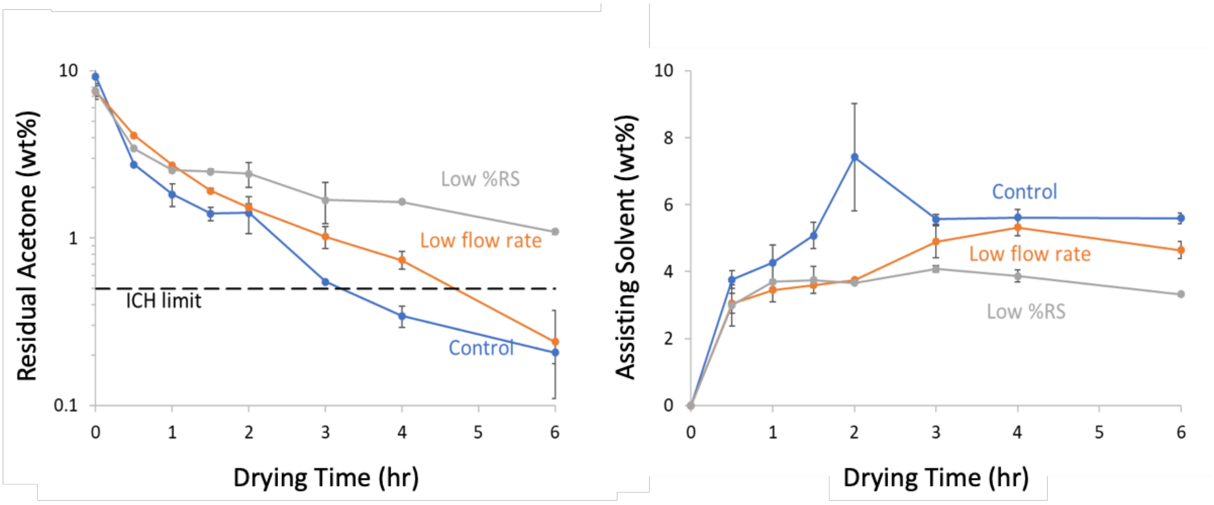

Figure S2. Drying curves for acetone removal from PMMAMA via methanol-assisted secondary drying at three process conditions (left) and uptake of assisting methanol during acetone removal from PMMAMA methanol-assisted secondary drying at three process conditions (right).

Methanol-assisted drying Experiments 1 and 4 (Table 2) were conducted at nearly identical conditions approximately 1 year apart. As Figure S3 demonstrates, the residual acetone removal in these two trials was nearly identical.

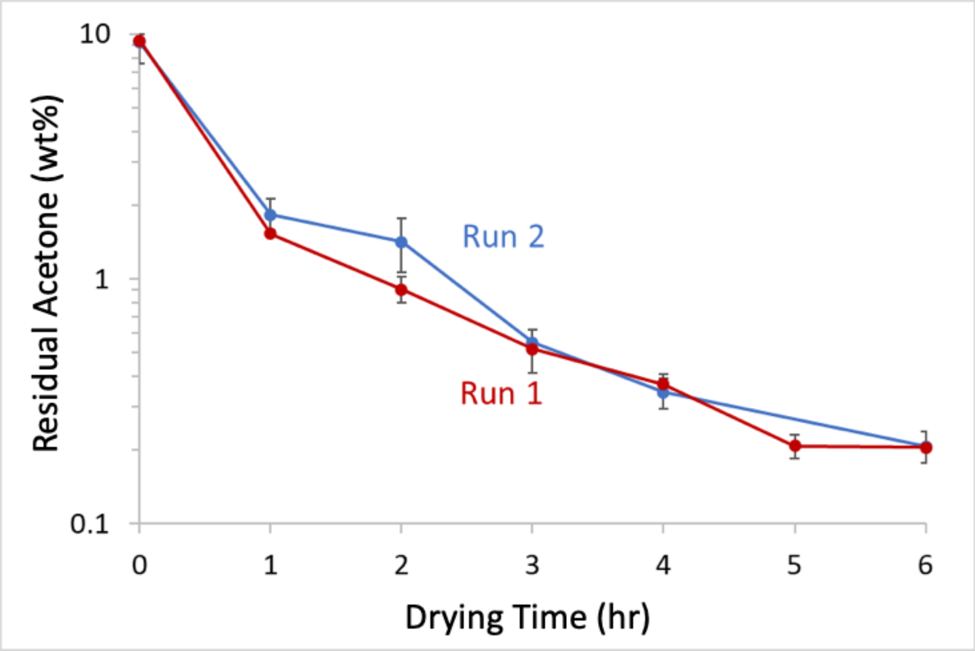

Figure S3. Residual acetone removal from trials run 1 year apart
